# Coronal plane alignment of the knee (CPAK) phenotypes and their relationship with gap patterns in robotic-assisted total knee arthroplasty: a clinical trial

**DOI:** 10.1007/s00132-026-04797-x

**Published:** 2026-03-24

**Authors:** Filippo Migliorini, Luise Schäfer, Jens Schneider, Andrea Maria Nobili, Daniel Kämmer, Nicola Maffulli, Andreas Bell

**Affiliations:** 1 Department of Trauma and Reconstructive Surgery, University Hospital of Halle, Martin-Luther University Halle-Wittenberg, Ernst-Grube-Street 40, 06097 Halle (Saale), Germany; 2 Department of Orthopaedic and Trauma Surgery, Eifelklinik St. Brigida, Kammerbruchstr. 8, 52152 Simmerath, Germany; 3 Department of Life Sciences, Health, and Health Professions, Link Campus University, Via del Casale di San Pio V, 00165 Rome, Italy; 4 Department of Trauma and Orthopaedic Surgery, Faculty of Medicine and Psychology, University La Sapienza, 00185 Rome, Italy; 5 School of Pharmacy and Bioengineering, Faculty of Medicine, Keele University, ST4 7QB Stoke on Trent, UK; 6 Centre for Sports and Exercise Medicine, Barts and the London School of Medicine and Dentistry, Mile End Hospital, Queen Mary University of London, E1 4DG London, UK

**Keywords:** Gap balancing, Robotics, Ausrichtung, Gelenkllinienorientierung, Gap-Ausgleich, Planning, Bandspannung, Biomechanik

## Abstract

**Background:**

Achieving optimal soft-tissue balance remains a key challenge in total knee arthroplasty (TKA), even with robotic assistance. The coronal plane alignment of the knee (CPAK) classification provides a structured description of constitutional alignment phenotypes, yet its relationship with intraoperative gap behavior has not been fully defined.

**Methods:**

A prospective series of 285 patients undergoing robotic-assisted TKA with the CORI system was analyzed. Knees were categorized according to the CPAK classification and intraoperative medial and lateral gaps were automatically recorded in extension (0°) and flexion (90°). Gap configurations were defined as balanced, varus, or valgus. Associations between CPAK phenotypes and gap types were assessed using χ^2^and logistic regression analyses, with CPAK IV serving as the reference phenotype.

**Results:**

The CPAK I and CPAK II phenotypes showed a significantly higher likelihood of varus gaps in both extension (odds ratio, OR 5.41 and 3.38, respectively) and flexion (OR 1.58 and 2.20, respectively), whereas CPAK VII was protective in extension (OR 0.23). Even valgus-aligned phenotypes demonstrated varus-dominant behavior in flexion. These findings indicate that gap behavior is strongly influenced by CPAK phenotype rather than solely by surgical alignment.

**Conclusion:**

Intraoperative soft-tissue balance during TKA reflects the underlying CPAK phenotype. Integrating CPAK classification into robotic planning enables anticipation of gap tendencies and facilitates phenotype-driven balancing, bridging constitutional anatomy with functional alignment.

**Graphic abstract:**

**Figure:**
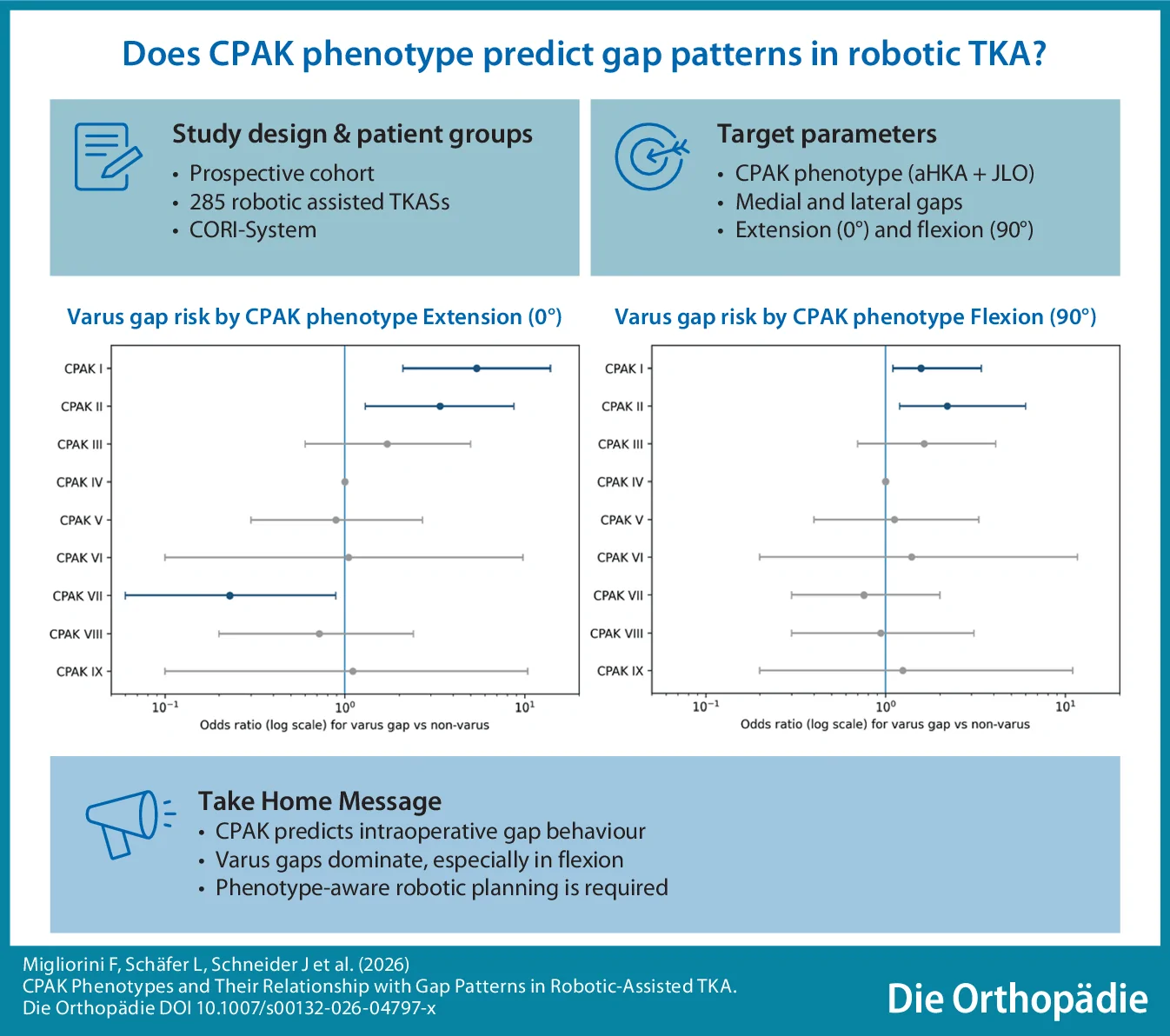

## Abstract (de)

**Hintergrund:**

Das Erreichen eines optimalen Weichteilgleichgewichts bleibt auch mit roboterassistierter Unterstützung eine zentrale Herausforderung bei der Implantation einer Knietotalendoprothese (TEP). Die Einteilung der koronalen Ausrichtung des Knies („coronal plane alignment of the knee“, CPAK) bietet eine strukturierte Beschreibung der konstitutionellen Alignment-Phänotypen, deren Zusammenhang mit dem intraoperativen Gap-Verhalten ist jedoch noch nicht vollständig geklärt.

**Methoden:**

Eine prospektive Serie von 285 Patienten, die sich einer robotergestützten TEP mit dem CORI-System unterzogen, wurde analysiert. Die Kniegelenke wurden gemäß der CPAK-Klassifikation kategorisiert; intraoperative mediale und laterale Gaps wurden automatisch in Streckung (0°) und Flexion (90°) aufgezeichnet. Die Spaltkonfigurationen wurden als ausgeglichen, Varus oder Valgus definiert. Die Zusammenhänge zwischen CPAK-Phänotypen und Spaltarten wurden anhand von χ^2^-Tests und logistischen Regressionsanalysen bewertet, wobei CPAK IV als Referenzphänotyp diente.

**Ergebnisse:**

Die Phänotypen CPAK I und CPAK II zeigten eine signifikant höhere Wahrscheinlichkeit für einen Varusspalt sowohl in Streckung (Odds-Ratio [OR] 5,41 bzw. 3,38) als auch in Flexion (OR 1,58 bzw. 2,20), während CPAK VII in Streckung eine schützende Wirkung hatte (OR 0,23). Selbst Valgus-ausgerichtete Phänotypen zeigten in Flexion ein Varus-dominantes Verhalten. Diese Ergebnisse deuten darauf hin, dass der Spalt (Gap) stark vom CPAK-Phänotyp und nicht ausschließlich vom chirurgischen Alignment beeinflusst wird.

**Schlussfolgerung:**

Der Ausgleich des Weichgewebes während einer TPE spiegelt den zugrunde liegenden CPAK-Phänotyp wider. Durch die Integration der CPAK-Klassifikation in die roboterassistierte Operationsplanung lassen sich Gap-Tendenzen vorhersagen und ein phänotypbasiertes Gleichgewicht erzielen, wodurch eine Brücke zwischen konstitutioneller Anatomie und funktionellem Alignment geschlagen wird.

## Introduction

Total knee arthroplasty (TKA) has long been recognized as an effective intervention for end-stage osteoarthritis [1–4], yet the pursuit of optimal alignment and soft tissue balance remains a central challenge. Traditionally, mechanical alignment was regarded as the gold standard, seeking to position the prosthetic components perpendicular to the mechanical axis to achieve symmetrical load distribution [5, 6]; however, this philosophy often disregarded the patient’s constitutional anatomy and natural soft tissue envelope, leading to altered kinematics and increased dissatisfaction in a subset of patients [5, 7, 8]. Kinematic alignment emerged as a paradigm shift towards restoring the individual’s prearthritic joint line orientation and limb morphology, aiming to reproduce the physiological laxity patterns inherent to each knee [9, 10]. Despite the conceptual appeal, achieving reproducibility and stability within these patient-specific frameworks has proven difficult, particularly given the wide interindividual variability in coronal alignment phenotypes [11–13]. The coronal plane alignment of the knee (CPAK) classification was developed to systematize this variability, integrating both the arithmetic hip-knee-ankle angle (aHKA) and joint line obliquity (JLO) to define nine distinct phenotypes [14–16]. These phenotypes provide a structured approach to understanding native coronal alignment, yet their intraoperative implications for gap behavior during TKA remain largely unexplored [17]. A deeper understanding of how CPAK phenotypes influence intraoperative balancing could bridge the gap between alignment theory and practical surgical execution.

Although several alignment strategies have been proposed in TKA, ranging from mechanical to kinematic or restricted kinematic philosophies, achieving a stable and well-balanced construct remains a key challenge [5, 9, 15, 16]. Intraoperative soft tissue balance is influenced not only by the surgical technique but also by the constitutional alignment phenotype of the knee [18]. The CPAK classification offers a reproducible framework for identifying these phenotypes, yet its direct relationship with gap patterns during surgery has not been fully elucidated [18, 19]. Clarifying this relationship could provide clinically relevant insights, allowing surgeons to anticipate soft tissue balancing challenges according to preoperative alignment type and to personalize alignment targets accordingly.

Therefore, the aim of this study was to evaluate the association between CPAK phenotypes and intraoperative gap characteristics in both extension and flexion. The underlying hypothesis was that CPAK phenotypes are not uniformly distributed in terms of balancing behavior and that specific subtypes, particularly varus-related configurations, are more likely to present varus gap tendencies intraoperatively. It was hypothesized that distinct CPAK phenotypes would demonstrate differential tendencies towards varus, valgus or balanced gap configurations and that varus-dominant CPAK phenotypes in particular would be strongly associated with intraoperative varus gap patterns.

## Methods

### Study design

All patients who underwent a robotic TKA at the Department of Orthoaedic and Trauma Surgery, Eifelklinik St. Brigida, Simmerath (Germany) from 2022–2025 were prospectively invited to participate in this investigation. The institution where the surgeries are performed is accredited by “EndoCert” (EndoCert certificate, Centres of German Endoprosthetic, German Society for Orthopaedics and Traumatology) [20], which supervises and certifies the quality of the surgical procedures. This study was conducted in accordance with the principles of the Declaration of Helsinki and its subsequent amendments. Ethical approval was granted by the North Rhine Medical Council, Düsseldorf, Germany (ID 2022374).

### Eligibility criteria

The inclusion criteria included being older than 18 years, able to provide informed consent and having symptomatic knee osteoarthritis graded stages II–IV using the Kellgren-Lawrence classification. The exclusion criteria consisted of: the presence of acute or chronic inflammatory diseases, cancer, pregnancy or breastfeeding, uncontrolled clotting disorders, abnormal blood cell counts, severe peripheral neuropathy, vascular diseases, peripheral ulcers, missing data related to the study’s endpoints, blood tests performed on days other than postoperative days (POD) 1 or 5, an inability to comply with the postoperative management protocol or any other condition that could influence study outcomes.

### Surgical technique

All patients underwent identical clinical, imaging and anesthesia protocols before and after surgery. Each patient received a single intravenous dose of 1.5 g cefuroxime at anesthesia induction and pain was managed with a continuous femoral nerve block maintained for 48 h. Every surgery was performed using the same standard medial parapatellar approach with functional kinematic alignment. All surgeries were performed using the CORI Robotic System (Smith & Nephew, London, UK). All implant components (Smith & Nephew Legion Genesis II, posterior-stabilized, polyethylene liner) were placed according to the manufacturer’s guidelines. Palacos cement was used for both femoral and tibial fixation. At the end of the surgery, 1 g of tranexamic acid was injected intra-articularly and two drainage systems were used: a deep intra-articular drain without suction and a superficial subcutaneous drain with suction, which remained in place for 48 h postoperatively. Enoxaparin sodium (40 mg/0.4 mL) was initiated 12 h postoperatively and administered daily for 6 weeks as antithrombotic prophylaxis. Standardized physiotherapy protocols were followed, with physiotherapists providing care starting from the first POD. If there were no complications or other medical reasons, the minimum hospital stay was 5 days; this was extended if patients experienced postoperative setbacks, such as reduced knee mobility, difficulty walking, or trouble with physiotherapy exercises. Starting on the second POD, patients participated in two daily physiotherapy sessions involving 60 min of continuous passive motion to increase knee flexion and extension, with the therapist progressively improving the range of motion. Discharge was based on achieving at least 80° of flexion. Ambulation began under supervision from POD 2, and stair use started on POD 4. Each patient then received a tailored rehabilitation program lasting at least 3 weeks, either as an inpatient or an outpatient. Any deviation from the planned surgical or rehabilitation approach resulted in exclusion from the study.

### Outcomes of interest

The primary outcomes of interest were the intraoperative extension and flexion gaps, assessed for both the medial and lateral compartments. All measurements were automatically recorded by the CORI robotic-assisted system at 0° (extension) and 90° (flexion) following osteophyte removal, bony resection, and soft tissue release. The system provided real-time quantitative values (mm) for the medial and lateral sides separately, enabling the identification of the intraoperative gap pattern. Based on the difference between lateral and medial compartments, gap configurations were categorized into three phenotypes: balanced (difference ≤ 2 mm), varus gap (lateral > medial by > 2 mm) or valgus gap (medial > lateral by > 2 mm). This definition was applied consistently in both extension and flexion. A mediolateral gap imbalance of no more than 2 mm in both full extension and at 90° of flexion has been suggested as a clinically relevant benchmark for intraoperative soft tissue balancing in TKA. Imbalances exceeding this margin have been linked to disturbed tibiofemoral kinematics, increased anterior tibial translation and poorer patient-reported outcomes [21–23].

Secondary outcomes included demographic and radiographic parameters to enable integration with the CPAK classification. The arithmetic hip-knee-ankle angle (aHKA) was calculated as the difference between the mechanical medial proximal tibial angle (mMPTA) and the mechanical lateral distal femoral angle (mLDFA). The joint line obliquity (JLO) was defined as the sum of mMPTA and mLDFA. According to the original CPAK classification, patients were stratified into nine phenotypes (I–IX) based on the combination of varus/neutral/valgus alignment (aHKA) and distal/neutral/proximal JLO. For each CPAK phenotype, intraoperative gap phenotypes were assessed in extension and flexion. Exploratory outcomes comprised associations between CPAK phenotypes and intraoperative gap distributions. In particular, the study sought to identify whether specific CPAK subtypes were associated with increased likelihood of varus or valgus imbalance compared with balanced patterns. Additionally, the concordance or divergence between extension and flexion gap profiles within each CPAK phenotype was explored to evaluate whether the same constitutional alignment predisposed to consistent or divergent balancing behavior across the motion.

### Statistical analysis

All statistical analyses were performed by the main author (F.M.) using IBM SPSS Statistics (version 25, IBM Corp., Armonk, NY, USA) and Stata (version 14, StataCorp, College Station, TX, USA). Continuous variables are expressed as means ± standard deviation (SD) or medians (interquartile range, IQR), depending on the distribution, while categorical variables are summarized as counts and percentages. Normality was assessed using the Shapiro-Wilk test. Intergroup comparisons of continuous data were performed using one-way ANOVA or the Kruskal-Wallis test, with a Bonferroni correction for post hoc pairwise analyses, as appropriate. Associations between categorical variables (CPAK phenotype vs. gap phenotype in extension or flexion) were evaluated using χ^2^-tests or Fisher’s exact test when cell counts were small; Cramer’s V quantified effect size. To estimate the magnitude of association, binary logistic regression models were constructed using the gap phenotype dichotomized as varus gap vs. non-varus (balanced or valgus). Given the limited number of cases in some CPAK categories, regression estimates for rare phenotypes were considered exploratory and were not used to draw definitive conclusions. The CPAK IV was selected as the reference phenotype as it represents the closest approximation to neutral coronal alignment. Odds ratios (OR) with 95% confidence intervals (CI) and *p*-values were reported for each CPAK subtype, both in extension and flexion. Furthermore, to account for the multinomial nature of gap phenotypes (balanced, varus, valgus), multinomial logistic regression models were fitted to estimate the predicted probabilities of each gap class for every CPAK phenotype. Model discrimination and calibration were assessed via Nagelkerkeʼs R^2^, classification accuracy and receiver operating characteristic (ROC) curves for binary comparisons (varus vs. non-varus). Interaction terms were tested to examine effect modification by sex, body mass index (BMI) and age. All statistical analyses were two-tailed, with *p* < 0.05 considered significant.

## Results

### Patient demographics

A total of 285 patients were included in the present analysis. The mean age was 67.6 ± 8.0 years (range, 46–87 years), and the mean BMI was 29.5 ± 4.4 kg/m^2^ (range, 19.5–43.4 kg/m^2^). The study cohort comprised 167 women (58.6%) and 118 men (41.4%). Laterality was evenly distributed, with 146 procedures performed on the left knee (51.2%) and 139 on the right knee (48.8%).

### Distribution of CPAK phenotypes

**Fig. 1:**
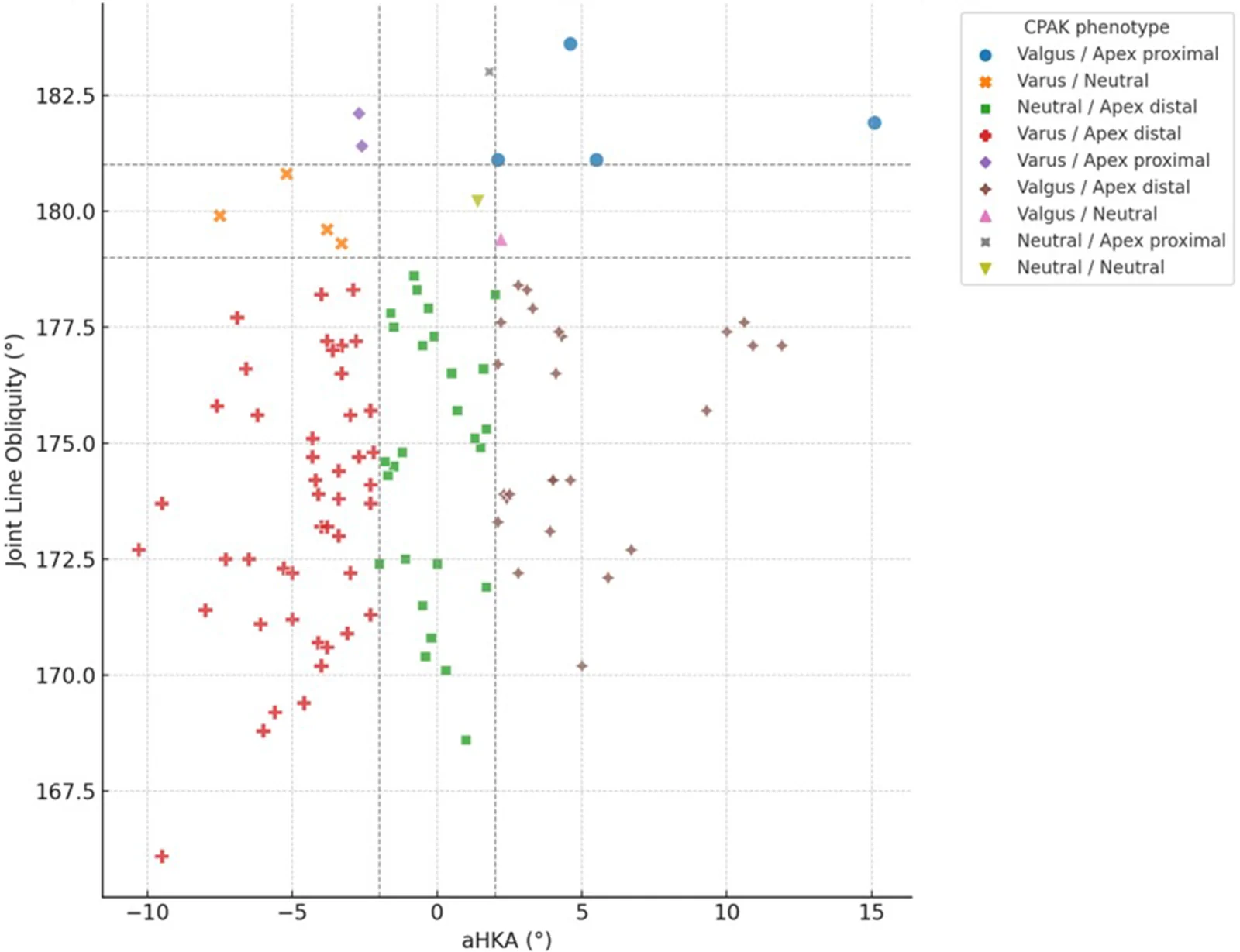
Scatter plot illustrating the distribution of patients across the CPAK phenotypes. The x‑axis represents the arithmetic aHKA and the y‑axis the JLO. Vertical dashed lines indicate the varus/neutral/valgus thresholds (−2° and +2° for aHKA), while horizontal dashed lines indicate distal/neutral/proximal joint line obliquity (179° and 181° for JLO). Each point represents one knee, color- and symbol-coded according to the CPAK phenotype

Patients were distributed across all nine CPAK phenotypes. The majority of knees were classified as CPAK I (*n* = 117, 41.1%), CPAK II (*n* = 70, 24.6%), and CPAK III (*n* = 67, 23.5%). Less frequent phenotypes included CPAK IV (*n* = 10, 3.5%) and CPAK IX (*n* = 10, 3.5%). Rare phenotypes were CPAK V, VI, and VIII (*n* = 3 each, 1.1%), while CPAK VII was observed in 2 cases (0.7%). Overall, this distribution reflects a clear predominance of varus-related CPAK phenotypes within the present cohort (Fig. 1).

### Association between CPAK phenotypes and intraoperative gaps

In extension, 79% of knees classified as CPAK I demonstrated a varus gap configuration, whereas 56% of CPAK III knees were balanced and 31% showed a valgus gap. The CPAK II group was more heterogeneous, with approximately half of the cases balanced and 39% presenting a varus gap. A strong overall association between CPAK phenotype and extension gap profile (χ^2^ = 75.6, df = 16, *p* < 0.000001, Cramer’s V = 0.62) was confirmed by χ^2^-testing.

In flexion, varus gaps predominated across most CPAK phenotypes. Notably, CPAK IV and VII demonstrated the highest likelihood of varus imbalance (84% and 79%, respectively). Even among valgus phenotypes, varus gaps were common in flexion, with CPAK III knees showing 64% varus configuration despite exhibiting valgus or balanced profiles in extension. The association between CPAK phenotype and flexion gaps was statistically significant, although less pronounced than in extension (χ^2^ = 33.7, df = 16, *p* = 0.0056, Cramer’s V = 0.34). Notably, this varus predominance in flexion was also observed in valgus-aligned CPAK phenotypes, indicating a convergence towards medial tightness despite neutral or valgus behavior in extension.

### Predictive analyses of gap profiles

Binary logistic regression (Table 1) showed that compared with CPAK II (reference), the odds of a varus gap were significantly higher for CPAK I (extension OR 5.41, 95% CI 2.1–13.9, *p* < 0.001; flexion OR 1.58, 95% CI 1.1–3.4, *p* = 0.02) and for IV (extension OR 3.38, 95% CI 1.3–8.7, *p* = 0.01; flexion OR 2.20, 95% CI 1.2–6.0, *p* = 0.03). Conversely, CPAK III was protective in extension (OR 0.23, 95% CI 0.06–0.89, *p* = 0.03), indicating a reduced probability of varus imbalance. Multinomial regression confirmed these findings, showing that CPAK I and CPAK IV had the highest predicted probability of varus gaps (≈ 75–80% in extension; 76–84% in flexion), while CPAK III carried the greatest valgus risk (≈ 31% in extension). Valgus gaps were otherwise rare (< 15% in all groups).

**Table 1:** Binary logistic regression for risk of varus gap across CPAK phenotypes

CPAK	Extension OR (95% CI)	*p*-value (ext)	Flexion OR (95% CI)	*p*-value (flex)	Clinical interpretation
1	5.41 (2.1–13.9)	< 0.001	1.58 (1.1–3.4)	0.02	Strong varus tendency in both extension and flexion
2	3.38 (1.3–8.7)	0.01	2.20 (1.2–6.0)	0.03	Persistent varus imbalance, especially in flexion
3	1.72 (0.6–5.0)	0.21	1.64 (0.7–4.1)	0.18	Tendency to varus, not statistically significant
4	Reference	–	Reference	–	Baseline comparator phenotype
5	0.89 (0.3–2.7)	0.75	1.12 (0.4–3.3)	0.81	Relatively balanced, no clear varus/valgus shift
6	1.05 (0.1–9.8)	0.96	1.40 (0.2–11.7)	0.76	Small numbers, no meaningful difference
7	0.23 (0.06–0.89)	0.03	0.76 (0.3–2.0)	0.58	Protective in extension, less prone to varus imbalance
8	0.72 (0.2–2.4)	0.61	0.94 (0.3–3.1)	0.92	Stable profile, no significant shift
9	1.11 (0.1–10.4)	0.92	1.25 (0.2–11.0)	0.85	Rare phenotype, inconclusive effect

*OR* odds ratios; *CI* 95% confidence intervals, *ext* extension, *flex* flexion, *CPAK* coronal plane alignment of the knee

## Discussion

The findings of the present investigation demonstrated a clear and consistent relationship between constitutional alignment phenotypes, as defined by the CPAK classification, and intraoperative soft tissue balance during robotic-assisted total knee arthroplasty. These findings suggest that intraoperative balance is largely constrained by constitutional phenotype rather than corrected by alignment alone, even in a robotic environment. The CPAK I and CPAK II phenotypes exhibited a strong predisposition towards varus gaps in both extension and flexion, confirming that knees classified within varus-related CPAK categories tend to maintain residual medial tightness even after bone cuts and standard releases. This behavior could be attributed to chronic soft tissue adaptation in long-standing varus CPAK configurations, where the medial collateral ligament and posteromedial capsule undergo progressive shortening and increased stiffness. Conversely, the CPAK VII demonstrated a protective pattern in extension, often achieving balanced or mild valgus configurations, possibly reflecting the greater elasticity and adaptive lengthening of lateral stabilizers in valgus CPAK profiles. Interestingly, even valgus-aligned CPAK categories did not exhibit intraoperative valgus imbalance in flexion, suggesting that flexion gaps are more strongly governed by posterior condylar geometry and tibial slope rather than static coronal plane phenotype. These observations align with previous evidence showing that flexion behavior tends to converge towards a varus-dominant pattern irrespective of preoperative alignment, supporting the hypothesis that kinematic constraints of the posterior compartment and femoral rollback mechanics inherently favor a medialized load distribution [24–26]. This finding is particularly relevant as it demonstrates that flexion gap behavior converges towards a varus-dominant configuration irrespective of the coronal phenotype. Even knees with valgus constitutional alignment exhibited medial constraint in flexion, suggesting that posterior compartment mechanics and femoral rollback exert a stronger influence than static coronal alignment.

From a biomechanical perspective, the predominance of varus gap behavior observed across multiple CPAK categories can be interpreted as the consequence of asymmetric load transmission and soft tissue tensioning intrinsic to the coronal phenotype. In CPAK varus phenotypes, the medial compartment acts as a fixed pivot subjected to higher compressive stress and reduced translation, whereas the lateral compartment displays greater excursion and lower constraint [27, 28]. Following arthroplasty, this asymmetry persists because prosthetic alignment correction restores the bony geometry but does not fully normalize the differential tension in periarticular structures [27, 29]. Furthermore, during flexion the lateral femoral condyle experiences posterior rollback along the sloped tibial plateau, producing greater lateral gapping while the medial compartment remains functionally constrained, thereby maintaining a residual varus moment [25]. The absence of true valgus imbalance even in CPAK valgus configurations reinforces the concept that intraoperative balance is dictated by the composite behavior of ligamentous tension, joint line orientation and femorotibial contact mechanics, rather than by coronal alignment alone [14, 30]. These findings suggest that CPAK classification captures not only static alignment characteristics but also the underlying biomechanical predisposition that influences the dynamic balance envelope during motion [30].

Clinically, these results highlight the relevance of integrating CPAK-based phenotyping into preoperative planning and intraoperative decision making. By anticipating the gap configuration associated with each CPAK category, surgeons can refine robotic alignment targets and minimize unnecessary soft tissue release, particularly in CPAK varus configurations where overrelease may induce instability or medial laxity [15, 27]. Within robotic workflows, CPAK classification provides a reproducible framework for anticipating intraoperative behavior, allowing the alignment philosophy to shift from geometric neutrality towards phenotype-driven functional restoration [15, 16]. This approach may reduce residual imbalance, optimize contact mechanics, and improve patient satisfaction by respecting the native biomechanics encoded within each CPAK phenotype [31]. Furthermore, the observed discordance between extension and flexion gaps across CPAK groups suggests that balancing strategies should adapt dynamically, as static preoperative alignment alone cannot fully predict intraoperative soft tissue response [32]. Robotic systems offer the advantage of quantifying these variations in real time, enabling surgeons to achieve a functional equilibrium tailored to the patient’s constitutional phenotype [15, 33, 34].

The present study has limitations that should be acknowledged. The predominance of varus CPAK phenotypes within the cohort reflects the natural distribution of knee osteoarthritis but limits the statistical generalizability of the results for rarer valgus categories. The investigation focused on intraoperative measurements without postoperative functional or radiographic correlation, thereby precluding conclusions about the long-term impact of CPAK-driven balancing on outcomes. Several CPAK phenotypes were represented by a limited number of patients, thereby limiting the statistical power of regression analyses for these categories. Therefore, results related to rare CPAK phenotypes should be regarded as exploratory and interpreted with caution. Nonetheless, the main conclusions of this study are supported by the most prevalent CPAK phenotypes, which showed consistent and clinically meaningful gap patterns. In addition, all procedures were performed using a single robotic platform and implant system, which may restrict applicability to other devices or alignment philosophies. Nevertheless, this study establishes a reproducible and biomechanically coherent association between CPAK phenotype and intraoperative gap pattern, providing a mechanistic foundation for future research integrating computational modelling, dynamic simulation and patient-reported outcome analysis to validate phenotype-guided alignment strategies in TKA. Finally, this study was intentionally designed as a mechanistic, hypothesis-generating investigation to elucidate the relationship between constitutional alignment phenotypes and intraoperative gap behavior. While postoperative functional outcomes were not assessed, the present findings provide a biomechanical framework upon which future outcome-driven and longitudinal studies can be built.

## Conclusion

Intraoperative gap behavior in robotic-assisted total knee arthroplasty is not random but phenotype-dependent. The CPAK classification offers a biomechanically meaningful framework for anticipating balancing patterns, particularly the varus predisposition observed across various categories. Integrating CPAK phenotyping into robotic planning enables surgeons to predict and individualize alignment strategies, bridging the gap between constitutional anatomy and functional restoration. Flexion gaps demonstrated a consistent varus tendency even in valgus phenotypes, underscoring the functional relevance of CPAK phenotyping.

## Data Availability

Data are available under reasonable request to the corresponding author.

## Abbreviations

aHKA: Arithmetic hip–knee–ankle angle
CI: Confidence intervals
CPAK: Coronal plane alignment of the knee
JLO: Joint line obliquity
mMPTA: Mechanical medial proximal tibial angle
mLDFA: Mechanical lateral distal femoral angle
OD: Odds ratios
POD: Postoperative day
SD: Standard deviation
TKA: Total knee arthroplasty

## Declarations

Ethical approval: Ethical approval was granted by the North Rhine Medical Council, Düsseldorf, Germany (ID 2022374). Registration: this study was not registered. Informed consent: all patients provided written consent to use their clinical and imaging data for research purposes.
